# A data set of sea surface stereo images to resolve space-time wave fields

**DOI:** 10.1038/s41597-020-0492-9

**Published:** 2020-05-15

**Authors:** Pedro Veras Guimarães, Fabrice Ardhuin, Filippo Bergamasco, Fabien Leckler, Jean-François Filipot, Jae-Seol Shim, Vladimir Dulov, Alvise Benetazzo

**Affiliations:** 1 France Energies Marines, Plouzané, France; 2 Univ. Brest, CNRS, IRD, Ifremer, Laboratoire d’Oceánographie Physique et Spatiale (LOPS), IUEM, Plouzané, France; 3 DAIS – Università Ca’ Foscari, Venice, Italy; 4 Service hydrographique et océanographique de la Marine (SHOM, HOM, REC), Brest, 29200 France; 5 KIOST – Korea Institute of Ocean Science and Technology, Busan, South Korea; 6 Marine Hydrophysical Institute, Sebastopol, Russia; 7 Institute of Marine Sciences, Italian National Research Council (ISMAR-CNR), Venice, 30122 Italy

**Keywords:** Space physics, Physical oceanography, Fluid dynamics

## Abstract

Stereo imaging of the sea surface elevation provides unique field data to investigate the geometry and dynamics of oceanic waves. Typically, this technique allows retrieving the 4-D ocean topography (3-D space + time) at high frequency (up to 15–20 Hz) over a sea surface region of area ~10^4^ m^2^. Stereo data fill the existing wide gap between sea surface elevation time-measurements, like the local observation provided by wave-buoys, and large-scale ocean observations by satellites. The analysis of stereo images provides a direct measurement of the wavefield without the need of any linear-wave theory assumption, so it is particularly interesting to investigate the nonlinearities of the surface, wave-current interaction, rogue waves, wave breaking, air-sea interaction, and potentially other processes not explored yet. In this context, this open dataset aims to provide, for the first time, valuable stereo measurements collected in different seas and wave conditions to invite the ocean-wave scientific community to continue exploring these data and to contribute to a better understanding of the nature of the sea surface dynamics.

## Abstract (summary)

**Table:** 

Measurement(s)	stereo image • wave height • peak wave period • wave direction
Technology Type(s)	Camera Device • computational modeling technique
Factor Type(s)	geographic location
Sample Characteristic - Environment	sea surface layer
Sample Characteristic - Location	Black Sea • Adriatic Sea • Yellow Sea • Iroise Sea

Machine-accessible metadata file describing the reported data: 10.6084/m9.figshare.12181158

## Background & Summary

Stereo imaging measurement of the sea surface elevation is based on single snapshots or time records captured by a pair of synchronized and calibrated cameras. The first projects to prospect the stereo photography use to measure sea surface topography was presented by^1,2^. However, the significant computational time required to extract the three-dimensional (3-D) elevation maps from a pair of images have limited the use of this technique until late 70s and early 80s with the works of^3,4^. In 1988^5^, used a pair of cameras mounted on an oceanographic offshore tower to measure the 3-D sea surface elevation to observe the directional distribution of short-scale ocean waves. Later on^6^, applied similar stereographic measurement techniques to study the 2-D wavenumber spectrum of short gravity waves.

More recently^7^, describe a stereo vision technique to measure the water surface topography. They used a conventional stereographic technique algorithms to survey geodetic surfaces and static objects. Based on that^8^, proposed a partially supervised technique (Wave Acquisition Stereo System, WASS) to estimate the 3-D shape of water waves using video image analysis with high spatial and temporal resolutions. Despite the basic principle remains more or less the same^9^, optimized the^8^ technique and presented the first open source code version of WASS (http://www.dais.unive.it/wass/).

Since the work by^8^, WASS and others similar stereo imaging systems have been widely used for different investigation purposes on ocean waves. For example^10–12^: used the foam footprint in the surface reflection to identified wave breaking in the video records, with that is possible observe the space and time evolution of breaking waves and investigate different properties of wave energy dissipation^13–15^,^16^ used stereo data to study the shape and likelihood of the highest waves, including maximum and rogue waves^17,18^; used the 3-D spectrum to explore non-linear waves properties like bound waves, second-order and harmonics, wave-current interactions and bi-modality. Stereo video system were also tested with certain level of success mounted over a moving vessels^12,19,20^ and^21^ also investigated its application in the surf zone.

As a general principle, stereo imaging results in a surface elevation map $\zeta (x,y,t)$ in the horizontal physical 2-D space ${\boldsymbol{x}}=(x,y)$ and time *t*. The coverage area, accuracy, and resolution depend mostly on the experimental setup (e.g. the distance between the cameras), camera specifications, and lenses^8,22^. See later in the paper a discussion about the expected errors for stereo observations. One of the greatest advantage of having the space-time elevation field $\zeta (x,y,t)$ is to compute the 3-D wavenumber/frequency spectrum without the need of invoking any (e.g. linear) wave theory for background noise removal and signal selection. This is most important for the short wave components, for which nonlinear contributions are more significant^6,17^.

This observation technique is very powerful to investigate many different process related to the sea surface and it brings a new perspective to investigate ocean waves, specially at mid to short scales. Stereo imaging connects the missing information between large to small-scale ocean observations (e.g., 10 m pixel resolution by SAR images) or a single point high frequency observation (wave gauge, moored buoys and others). In this context, stereo video have been prof to be useful to explore different aspects of ocean waves.

In this paper, for the first time, we aim at presenting a valuable stereo-image data set, free available for the scientific community. This data set contains original records, calibration files, configuration files for processing, and examples of post-processed surface elevation maps collected during several oceanic campaigns, at different locations around the world ocean and under different sea state conditions. All this makes this data set especially useful to explore a common framework to investigate ocean waves and the sea surface dynamics.

## Methods

The data set contains two main assets. First, we provide several raw stereo-image acquisitions, that consists in about 30 min of pair of images sequences of the sea surface, for different locations in the world oceans and met-ocean conditions. Such images are captured with two cameras, temporally synchronized and optically calibrated, to allow the 3-D geometrical analysis of the observed scene. Second, we processed the stereo sequences with the currently available state-of-the-art WASS tool to obtain space-time fields of wave elevation data that can be conveniently used for further analysis.

Due to their different nature, the two assets deserve a separate discussion on what concerns their description. The stereo images represent the *raw data* directly acquired from the observed phenomenon. Consequently, we will focus on the hardware side, describing the imaging technologies involved and, even more importantly, on the optical characteristics of the camera-lens system. On the other hand, the operations we performed to get the final space-time field is a pure algorithmic process that could potentially be improved in the future. As such, we will summarize what tools were used to process the data together with the raw stereo images. So any researcher is welcome to reprocess them based on its one personal interest and convenience, for example, by choosing a finer/larger interpolation grid, a different range of the gridding domain and technique to interpolate the data, or outlier filtering scheme. Therefore, we’ll concentrate on the kinds of errors that should be kept under observation at each stage of the processing, to guarantee that the final elevation model is an accurate measurement of the real imaged sea surface.

### Stereo images

Image data is grouped in several acquisitions (records), each one composed by a sequence of images (namely a *stereo sequence*) captured by two cameras. The two cameras are temporally synchronized such that, for each image acquired by the first, there correspond an image acquired by the second with a maximum delay in the order of microseconds. The synchronization is ensured by a cable connecting the two devices and a *hardware trigger* generating electrical impulses at a predefined frequency. The final result is, for each record, a sequence of several image pairs, acquired at a given rate (usually around from 10 to 15 Hz).

**Fig. 1:**
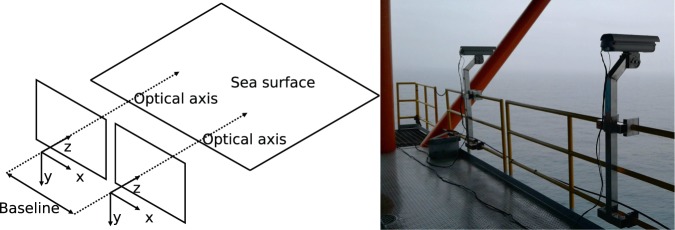
Example of stereo imaging system. Left: Geometric configuration of the stereo camera systems. Right: System installed on the Socheongho Ocean Research Station (Yellow Sea). The two cases holding the digital cameras are seen in the horizontal position used for alignment, before being inclined down-looking in their operative position.

Cameras are placed side-by-side, with parallel optical axes, both down-looking at the sea surface. The baseline (i.e. the vector connecting the two optical centers) spans the x-axis of the camera reference frames as shown in Fig. 1 (Left). The geometric setup is “loose”, in a sense that no mechanical solution was used to enforce it. For example, the optical axes were manually aligned just by visual inspecting the resulting images. We must stress, however, that each camera position must not be known precisely a-priori for the success of the reconstruction. Thus, the ideal arrangement in Fig. 1 should be considered as a guideline for a successful setup more than a strict operational requirement. The two cameras are referred as *Camera 0* and *Camera 1*, regardless their spatial configuration. Usually the *Camera 0* is placed at the right-hand side of *Camera 1* but this is not mandatory for the tools used for reconstruction (the spatial configuration can be automatically inferred from the extrinsic calibration performed directly on the acquired images).

In all the records we used a pair of BM-500GE JAI cameras, producing gray-scale images with a spatial resolution of 2048 × 2456 pixels (5 Megapixels) and intensity levels quantized to 8 bits (i.e. 256 different shades of gray). Exposure time was set to a value less then 0.005 s to avoid motion blurring and, if necessary, compensated by the analog gain control of the camera to obtain images in which the average intensity level of the pixel in a central Region of Interest (ROI) is ~110. Care was taken to ensure that both the stereo images exhibit the same average intensity level in their own ROI to maximize the photometric consistency of corresponding points (see next section for details).

**Fig. 2:**
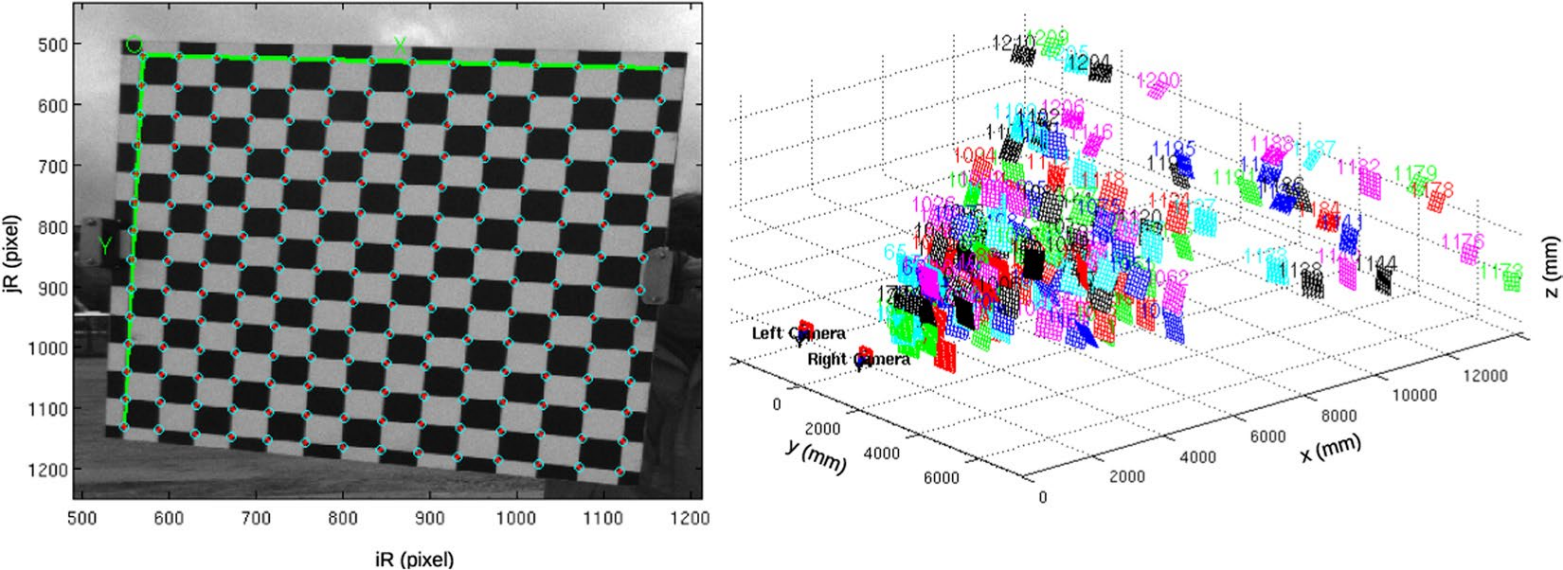
Example of stereo camera calibration. Left: chess board corners detection for intrinsic cameras calibration. Right: Chess board corners reconstruction in a real world coordinate system.

To reconstruct the metric properties of the observed scene, the optical model of each camera must be taken into account. We considered the common pinhole camera model with a 5-degrees polynomial radial distortion, resulting in a total of 9 parameters, namely: the *focal length* (2 parameters, to account for non-square pixels), the position of the *principal point* (2 parameters) and the 5 parameters used to model the *radial and tangential distortion*. To estimate them, the *intrinsic calibration* was performed on each camera before installation consisting in the acquisition of a known chessboard target in dozens of different poses (Fig. 2). We used the Camera Calibration Toolbox for Matlab, provided by Jean-Yves Bouguet (www.vision.caltech.edu/bouguetj), implementing the method described in^23^.

### 3-D Reconstruction and Wave Field estimation

Each record of the data set is composed by a sequence of stereo frames and the intrinsic calibration data for each camera-lens system. The process used to reconstruct the spatio-temporal wave field is divided in three subsequent steps. First, the relative spatial position of the two cameras (ie. the *extrinsic calibration*) is recovered automatically by analyzing a subset of stereo frames. Second, each stereo pair is processed to reconstruct a dense 3-D point cloud of the observed sea surface. Finally, each point cloud is filtered, cleaned and interpolated on a regular grid to produce a *data cube* containing the spatial surface elevation over time. We used the *WASS pipeline*^9^ for the first two-steps and Matlab^®^ scripts for the last one.

**Fig. 3:**
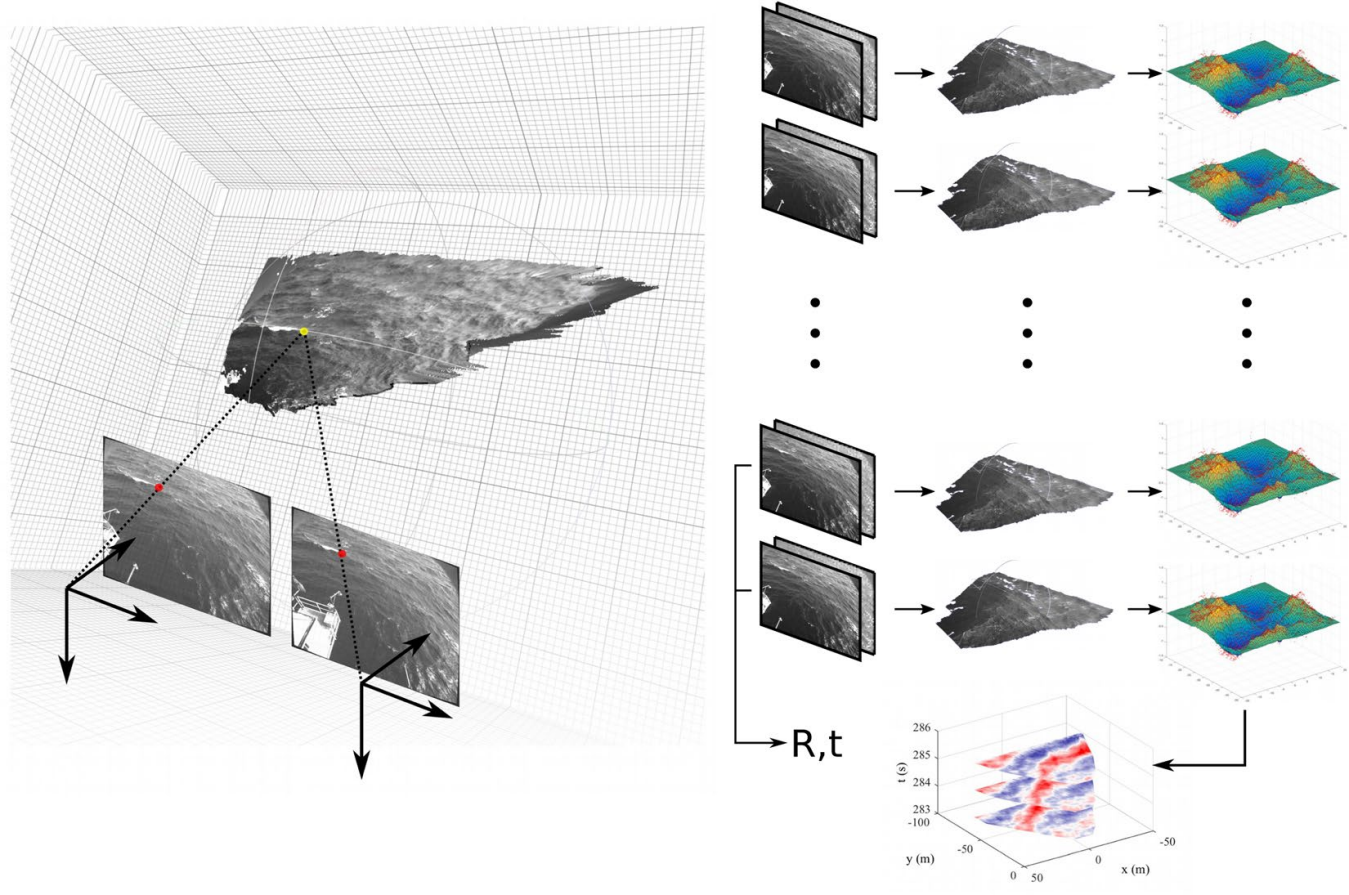
Left: 3-D surface points can be estimated by triangulating exiting rays passing through corresponding image points. Right: wave field estimation process. A subset of the stereo frames are used to compute the *extrinsic parameters* (**R**, $\overrightarrow{T}$) necessary to create a dense 3-D point cloud for each stereo-pair. Each point cloud is interpolated in a regular surface grid and a spatio-temporal wave field is obtained by stacking all the reconstructed surfaces.

The principle of stereo reconstruction is easy to understand. It is based on the assumption that we geometrically know the imaging model of the two cameras (*intrinsic parameters*) and their relative poses described by a rotation matrix ${\bf{R}}$ and a translation vector $\overrightarrow{T}$ (known as *extrinsic parameters*). In this scenario, once a pixel-to-pixel correspondence is established between a point on one camera and the same point on the other (i.e. the two projections of the same 3-D feature on a scene), the exiting rays of the two cameras can be intersected to recover the 3-D position of the point. This process is known as *triangulation* (See Fig. 3, Left). Stereo reconstruction algorithms usually exploit the *epipolar constraint* to limit the search space of corresponding points. A pre-processing step called *rectification* warps the two frames such that corresponding points lie on the same scan-line of each image as described in^24^.

Extrinsic parameters are estimated on the stereo images itself with a procedure known as auto-calibration. The idea is to find the best rigid motion (${\bf{R}},\overrightarrow{T}$) such that a set of point-to-point correspondences (determined by any robust matching function) satisfy the resulting epipolar constraint. Since the resulting epipolar lines are invariant with respect to the norm of $\overrightarrow{T}$, the camera pose can be estimated up to an unknown baseline distance that has to be measured in other ways. The *wass_match* and *wass_autocalibrate* programs are used to perform robust point matching and auto-calibration respectively. At the end of this step, the WASS pipeline provides the estimated (${\bf{R}},\overrightarrow{T}$) together with some statistics useful to evaluate the accuracy of the output. In particular, two parameters are important to be monitored: (i) the number of point-point matches that are considered inliers (i.e. valid) with respect to the estimated pose; (ii) the average epipolar error of the inlier matches (ie. how coherent are the inliers wrt. the epipolar constraint). In all our sequences we obtained hundreds of matches with an average epipolar error ranging from 0.3 to 0.5 pixels. Considered that the epipolar constraint is subject to just 5 degrees of freedom, we believe that such high number of correct matches is a good indication of an actual good calibration of the *extrinsic parameters*.

After the extrinsic calibration, the *wass_stereo* program is executed for each stereo pair to produce a dense point cloud. The number of reconstructed points vary with the scene but is ~3 × 10^6^ points (for 5 Megapixel cameras). A plane is fitted in a least-squares sense to each 3-D point cloud to produce an estimate of the mean sea plane position in the right camera reference frame. The accuracy of the reconstructed plane depends to the number of wave and crest lengths that are visible in the camera field of view^19^. To reduce the uncertainty, we take advantage of the quasi-Gaussian nature of the sea surface elevation to average the plane parameters among each record. The obtained average sea plane is then used to compute the transformation mapping the 3-D data from the camera to a geographic reference frame with the z-axis pointing upward with respect to the gravity vector. The details of this transformations are explained in^19^.

**Fig. 4:**
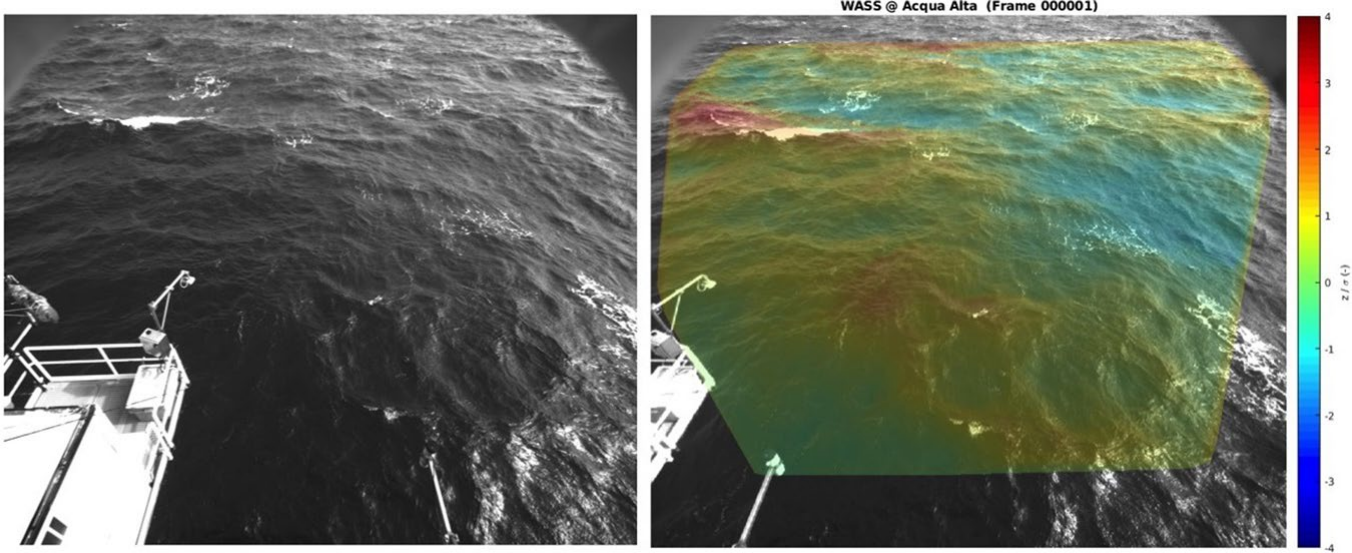
Example of stereo-image pair (from the Acqua Alta tower in the northern Adriatic Sea, Italy) and 3-D wave field on top of the right image (the colorscale is proportional to the sea surface elevation $\zeta$).

Each geographically-aligned point cloud is linearly interpolated on a regular horizontal 2-D grid to produce a discretized surface elevation map, which is generally used for wave analysis. In the results presented in this study, the $x$-axis is set along the cameras supporting bar (positive direction towards the right camera), and the y-axis points away from the cameras. If a sequence of image pairs was collected, the above-mentioned operations are repeated at each instant and the final result will be a (2-D + time) burst of sea surface elevations. An examples of a typical stereo-image pair and the measured 3-D surface geometry can be seen in Fig. 4.

### Experimental setup

**Fig. 5:**
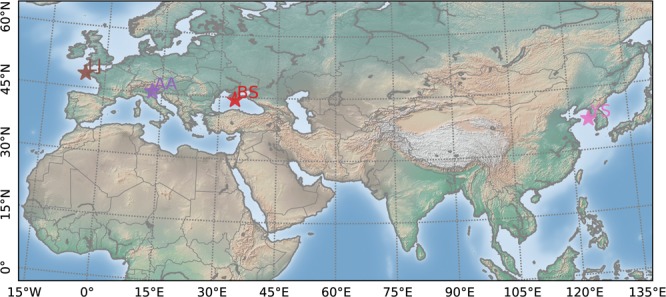
Location of the four stations with deployed stereo systems for oceanic wave observation. From west to east: La Jument lighthouse (LJ), the Acqua Alta tower (AA), the Marine Hydrophysical Institute (BS), and the Sogheongho Ocean Research Station (YS).

Each wave data set presented in this study was acquired using an experiment setup ad-hoc designed to best represent its initial research purpose. At the time of this publication, this data set contains twelve records: seven recorded at the Marine Hydrophysical Institute platform in the Black Sea; three recorded from the Acqua Alta oceanographic research tower in the Adriatic Sea; one recorded at the Sogheongho Ocean Research Station in the Yellow Sea; one recorded at La Jument lighthouse in the Iroise Sea. For each experimental setup, principal parameters are reported in the Table 1 and details of each station (see Fig. 5) are given in the following paragraphs.

**Table 1:** Stereo-image data sets: Record starting time; acquisition frame rate (fps); number of synchronized pair of images (# imag.); approximate record duration (*Durr*.); Left-to-right camera distance, baseline (*B*); Reference to explore the stereo set up and wave data (Ref.).

ID	Record (UTC)	fps (Hz)	# imag.	*Dur*. (min)	*B* (mm)	Ref.
BS 01	2011/10/01 16:18:00	15	10828	7	2030	^10^
BS 02	2011/10/04 11:38:00	12	14375	20	2030	^10^
BS 03	2011/10/04 13:07:00	12	21574	30	2030	^10^
BS 04	2011/10/04 15:30:00	12	21575	30	2030	^10^
BS 05	2013/09/22 13:00:01	10	17981	30	1872	^12^
BS 06	2013/09/25 12:15:01	12	21578	30	1872	^12^
BS 07	2013/09/30 10:20:01	12	21578	30	1872	^12^
AA 01	2014/03/27 09:10:00	12	43161	60	2545	^31^
AA 02	2015/03/05 10:35:00	12	21573	30	2545	—
AA 03	2015/05/15 09:00:00	12	21552	30	2545	—
YS 01	2017/05/13 05:00:00	10	6000	10	5040	^27^
LJ 01	2018/01/03 09:39:38	10	18000	30	5445	^28^

**Table 2:** Experimental conditions: significant wave height (*H*_*s*_); peak wave period (*T*_*p*_); peak wave direction (*D*_*p*_); estimated wind speed at 10-m height (*U*_10_); wind direction (WD); expected wave age (*C*_*p*_/*U*_10_) from deep water linear theory without current ${C}_{p}=g{T}_{p}/2\pi$; near-surface current speed (C) and direction (CD) from AWAC at AA platform and from X-band radar at LJ.

ID	*H*_*s*_ (m)	*T*_*p*_ (s)	*D*_*p*_	*U*_10_ (m/s)	WD	*C*_*p*_/*U*_10_	C (m/s)	CD (°*N*)
BS 01	0.30	3.03	WSW	10.7	WSW	0.44	—	—
BS 02	0.36	2.63	WSW	10.1	WSW	0.41	—	—
BS 03	0.45	3.03	SW	12.2	WSW	0.37	—	—
BS 04	0.55	3.70	SW	12.9	WSW	0.45	—	—
BS 05	0.66	4.30	E	8.7	E	0.77	—	—
BS 06	0.41	4.10	SW	6.1	N	1.05	—	—
BS 07	0.65	5.70	SW	15.2	N	0.58	—	—
AA 01	1.36	4.49	ENE	9.9	ENE	0.71	0.24	45
AA 02	1.99	6.56	ENE	12.9	ENE	0.80	0.13	47
AA 03	1.33	5.69	ESE	10.2	E	0.87	0.15	84
YS 01	1.94	6.70	SW	16.0	NW	0.65	—	—
LJ 01	10.03	13.33	W	14.7	W	1.41	0.92	323

**Table 3:** Expected root mean square error *RMS* and estimated high frequency limit *f*_*lim*_ for each experimental setup.

	*RMS*_*z*_	*RMS*_*x*_	*RMS*_*y*_	*f*_*lim*_(*Hz*)
BS 01–04	0.01 m	0.01 m	0.08 m	1.2
BS 05–07	0.01 m	0.01 m	0.08 m	1.4
AA 01–03	0.01 m	0.02 m	0.05 m	1.5
YS 01	0.03 m	0.05 m	0.17 m	0.9
LJ 01	0.09 m	0.14 m	0.35 m	0.5

**Fig. 6:**
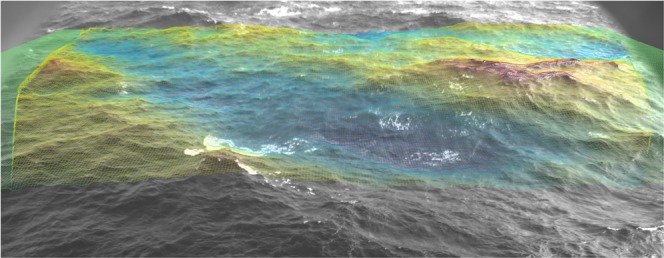
The 3-D surface grid computed by the reconstruction pipeline rendered on top of the original image captured by the right stereo camera. The rendering is produced by the provided *wassncplot* tool together with the mapping between image pixels and 3-D coordinates in the geographical reference frame of the grid.

**Fig. 7:**
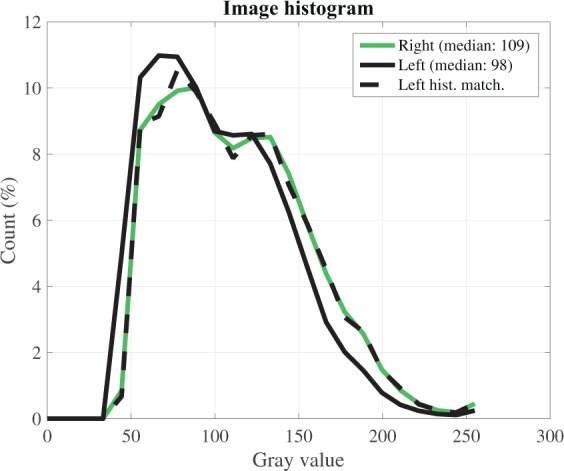
Example of left and right stereo image gray values histograms. The histogram of the left image transformed to match the right image is shown with a dashed black line.

**Fig. 8:**
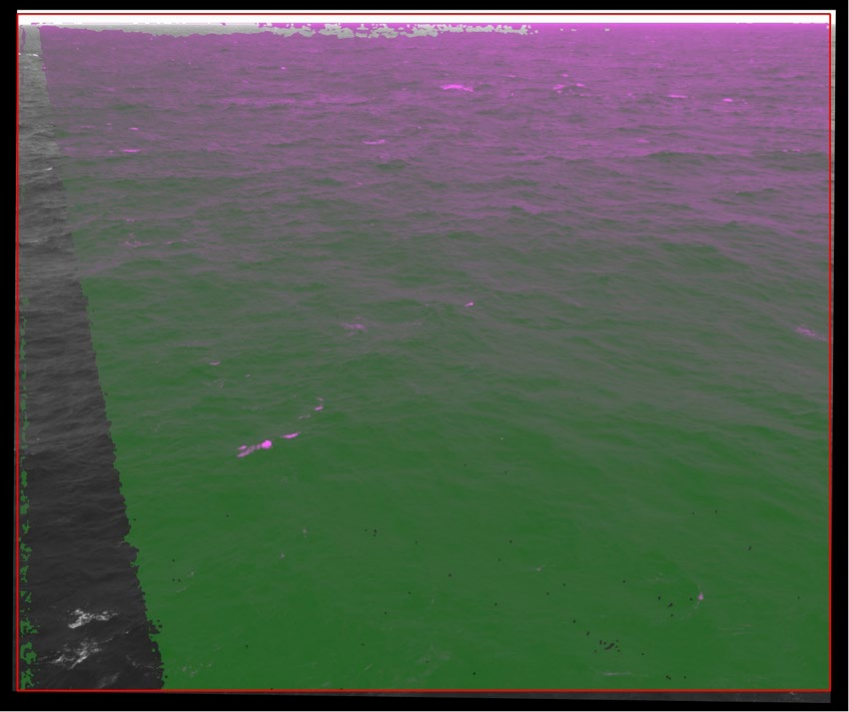
Example of matched pixels between left and right images.

**Fig. 9:**
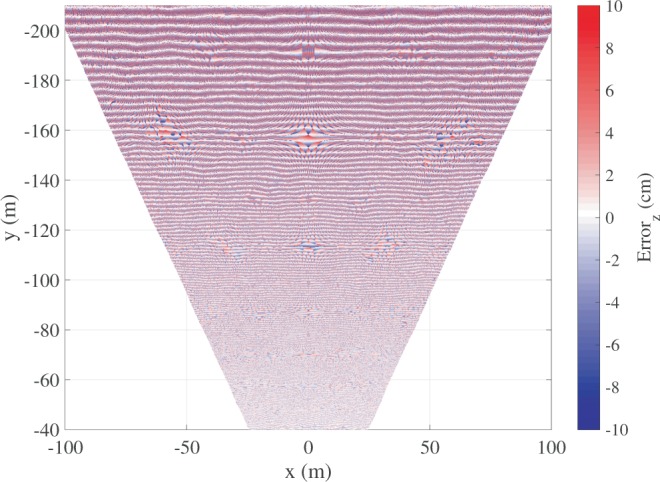
Example of the 2-D horizontal map of the vertical component $(er{r}_{z})$ of the quantization error within the field of view of the stereo cameras. Stereo system specifications: height of 33 m, baseline of 5 m, focal lens length 5 mm, 5 Megapixel cameras.

**Fig. 10:**
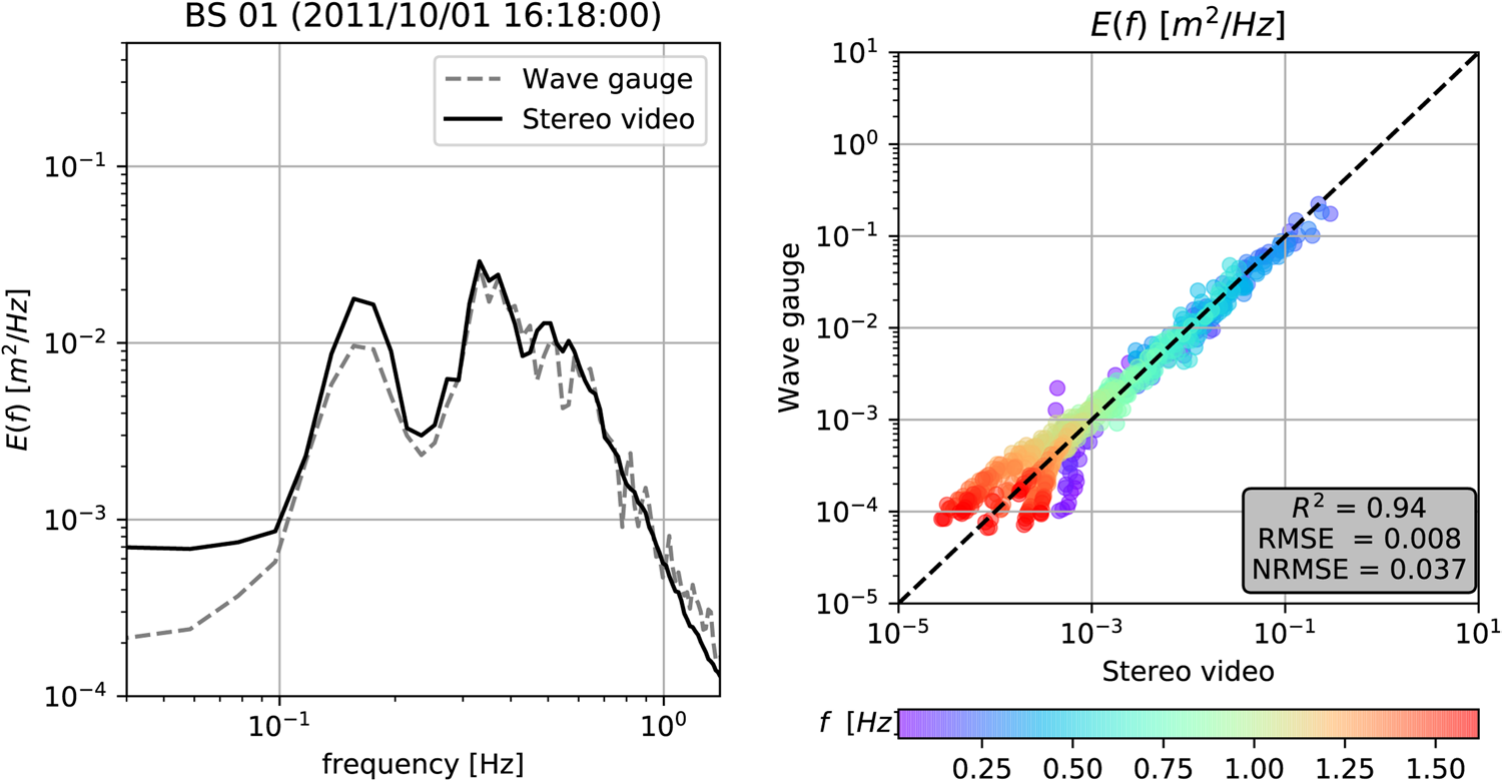
BS records collected in 2011 and 2013: frequency spectrum $E(f)$ estimated from the stereo system, compared with data from a wire wave gauge mounted on the platform. (Left) Example of BS 01 wave spectrum recorded by stereo video system compared with a wave gauge. The spectrum from stereo data is averaged over a 10.8-m side square analysis window, and thus the random sampling error is smaller for the shorter waves with many uncorrelated waves in the field of view. (Right) Comparison between wave gauge and stereo video wave spectrum for all BS records provided at this dataset. The color scale represents the wave frequency of that sampled data. In the gray box are shown the squared Pearson correlation coefficient (*R*^2^), the Root Mean Square Error (RMSE) and the Normalized Root Mean Square Error (NRMSE).

#### Black Sea (BS)

The Black Sea data set was acquired by a stereo imaging system mounted on the research platform of the Marine Hydrophysical Institute, 500 meters of the coast next to Katsiveli coast in the Black Sea (44.39°N, 33.98°E). The water depth at the observation area is about 30 m. The stereo video system was mounted 11 m above the sea surface and used a pair of synchronized 5 Megapixel (2048 × 2456 pixels) BM-500GE JAI cameras. The data set acquired in 2011 used cameras equipped with a 5-mm focal lens^10,17^. In 2013, to observe a larger area of the sea surface, it was used the same cameras but with wide-angle lenses^12^. The cameras were installed on the eastern corner of the deck of the platform, point to a direction 82° clockwise of North. Additionally, in the other side of the platform the sea surface elevation was also measured at a 10 Hz by six restive strings arranged in a centered pentagon of radius 25 cm recorded sea surface elevations with an accuracy of 2 mm providing the data for frequency-directional spectrum estimations in the rage of 0.1–1 Hz. A more detailed description of the platform instrumentation is given by^25^. The wind speed and direction, air temperature, humidity, pressure and water temperature are measured at the center of the platform, 23 m above the sea level by a “Davis Vantage Pro 2” meteorological station.

### Acqua Alta (AA)

The Acqua Alta stereo data set was collected using an imaging system installed on the north-east side of the Acqua Alta oceanographic research tower, located in the northern Adriatic Sea (Italy, 45.32°N, 12.51°E), 15 km off shore the Venice littoral (Italy). The Acqua Alta tower is managed by the Institute of Marine Sciences (ISMAR) of the Italian National Research Council (CNR) and it is the only manned platform in the Mediterranean Sea dedicated to the oceanographic research. On the tower stereo cameras were mounted at 12.5 m above the mean sea surface, looking to northeast (45°N clockwise) and the local water depth in front of the cameras view is about 17 m. The stereo imaging system also uses two BM-500GE JAI digital cameras mounting 5-mm focal length lenses (see^26^). The wind speed and direction are measured at the south-east corner of the tower, about 15 m above the sea level. Supplementary instrumentation (e.g. a Nortek AWAC) was continuously used to assess the stereo observations. More details about this setup can be found at^14^.

#### Yellow Sea (YS)

The Yellow Sea data were collected from the Socheongcho Oceanic Research Station (37°25′23.28″N 124°44′16.94″E), managed by the Korea Institute of Ocean Science and Technology. A couple of visible-range, synchronized digital cameras (8-bit grayscale, with 2456 × 2048 pixel resolution and 3.45 *μm* square active elements) mounting 8 mm distortion lenses, were placed 5.04 m apart and 33 m above the mean sea level on the east side of the station. The cameras set looking to east (90°N clockwise) and were rotated down-looking with an inclination angle of about 25°. At the station, the local depth is on average 50 m, the sea current spectrum is dominated by semi-diurnal tides, with a tidal ellipse having principal axis oriented northwest-southeast, and maximum speed along that direction in excess of 1 m/s. More details about this setup can be found at^27^.

#### La Jument (LJ)

La Jument, lighthouse is located offshore of Ushant island, Western Brittany, Iroise Sea, France (48°25′20.00″N 5°8′2.28″W). This lighthouse is extreme exposition to North East Atlantic storms waves and strong tidal currents. The waves that shoal over the very steep up-wave bathymetry can violently break over the lighthouse and generate sea spray that can go up well over the lighthouse lantern. The stereo video data was collected there is part of the DiMe project dealing with the characterization of extreme breaking waves. This lighthouse lies over a rocky platform that drops abruptly on its western boundary. The upper deck of the lighthouse is located at about 45 m above the mean sea level, but it is very sensitive to a macro-tidal conditions that can range up to 8 m. This makes possible the observation of very high sea states in intermediate to shallow waters with a large field of view and a minimal shadowing effect. The bathymetry around it decreases very sharply going toward the ocean. The depth at 50 m from the cameras view is about 30 m and goes to 60 m at 300 m away from the lighthouse. The video cameras were set near to the top of the lighthouse (about 45 m high) and looking at 246°N. The distance between the cameras were 5.445 m. The video system also the same 5 Megapixel BM-500GE JAI cameras with 5-mm wide angle lens (same as^12^, BS_2013 records). The system was set to 10 Hz sampling frequency. More details about this experiment and this lighthouse can be found at^28^.

## Data Records

This data set combines a sample of stereo records acquired at different locations, considering several sea state conditions and related to different research projects. However, the goal is to feed the data set with new records as soon as they will become available from new research campaigns at^30^. A summary of the main sea state characteristics of each experiment is presented in Table 2.

The data acquired at BS in 2011 (BS 01 to 04) is mostly composed by young wave propagating in the same wind direction. This data was used by^10^ to investigate the properties of short breaking waves and compared with the dissipation source function in the energy balance equation^17^ used BS 02 to 04 to investigate the energy level and its directional distribution to investigate non-linear wave interaction at frequencies up to 1.4 Hz and for wave length from 0.6 to 12 m. Based on the 3-D wavenumber/frequency spectrum $E({k}_{x},{k}_{y},f)$ they separate the linear waves from the bound second-order harmonics and found that the harmonics were negligible for frequencies *f* up to 3 times ${f}_{p}$, but account for most of the energy at higher frequencies. In the range $2{f}_{p}$ to $4{f}_{p}$, they observed that the full frequency spectrum decays like ${f}^{-5}$, which means a steeper decay of the linear spectrum. At BS 03^17^, observed a pronounced bi-modal direction distribution, with two peaks on either side of the wind direction, separated by 150° at $4{f}_{p}$.

The BS 05 to 07, were acquired in 2013 and used by^12^ to investigate the longer waves modulation impact on short wave breaking. Those acquisitions consists in bi-modal sea state conditions with short wave breaking. BS 05 consists in a case of longer and shorter wave propagating in the same direction (East). The longer wave was ${H}_{s}=0.4$ m with a peak at 4.25 s, while the local short wave system is dominated by 3 s waves from East and modal amplitude around 10 cm. BS 06 is characterized by longer and shorter waves propagating almost in orthogonal direction, with 0.4 m and 4 s swell and very short short wave driven by the local wind, with 1.8 s peak period and 10 cm amplitude. BS 07 occurs under strong northerly wind conditions, with an 0.65 m opposing swell of 5.6 s peak period from the south-west, 135° from the wind direction. The short wave spectrum has a 0.32 m wave height and 1.8 s peak period from North.

AA stereo images portray space-time wave fields generated by prevailing east northeasterly winds producing, generally, fetch-limited waves in the northern Adriatic Sea. The AA 01 record was acquired during a cross-sea condition, being the dominant wave components aligned with the local wind (ENE) while a swell was produced by southeasterly winds blowing in the central Adriatic Sea. Breaking events at different scales and directions are visible in the stereo images. Space-time wave fields show the presence of second-order harmonics and a bimodal distribution of the short-wave energy. These data were used to investigate the benefit of using stereo observations to calibrate the X-band marine radar MTF^31^. Wave elevations stored in the NetCDF4 file were low-pass filtered at 1.5 Hz to remove the influence of high-frequency quantization noise. AA 02 data were acquired during a ENE wind-forced condition. Wave breaking occur for dominant waves (along the peak direction) and for short waves (angled with respect the peak direction). Occasionally foam stripes aligned with the wind are detectable. AA 03 consists of easterly wave fields. Very few breakers are visible within the images.

YS data include wind-generated 3-D wave fields observed during the passage of an atmospheric front, which led to a wide directional spreading of wave energy. Two independent wave systems were detected in the data, clearly identified in the 3-D wavenumber/frequency spectrum $E({k}_{x},{k}_{y},f)$. Using this record^27^, examined the shape and the nonlinear properties of the wavenumber/frequency 3-D wave spectrum, and the characteristic spatial, temporal and spatio-temporal length scales of the wave field. Data were also used to analyze the probability of occurrence and the spatio-temporal size of rogue waves, in particular the extent of the horizontal sea surface region they span.

LJ 01 consist in our biggest sea state condition recorded by the moment, with individual wave heights up to 23.6 m. This case was recently used by^28^ to characterize extreme breaking waves and their mechanical loading on heritage lighthouses at sea. They focus their study in a particular wave breaking event with a crest elevation high enough to pass over the substructure and hit directly the lighthouse tower. This event was marked by a strong peak in the horizontal accelerations measured inside the light house and an extreme wave “runup” that reaches more than 42 m blinding the cameras lenses.

## Technical Validation

The key variables to be validated is the sea surface elevation derived with the stereo processing. Whenever possible, stereo data have been assessed with reference instrumentation expected to have at least the same performance as the imaging system. As many factors may influence the quality of the wave observations, we provide below the description of the principal ones providing the target values for an optimal data set. Five sources of uncertainty can impact the observations: the uncertainty in the internal parameter calibration (internal calibration error, $er{r}_{ic}$), the uncertainty in the external parameter calibration (external calibration error, $er{r}_{ec}$), the uncertainty in the determination of the corresponding pixels (matching error, $er{r}_{ma}$), the uncertainty in the recovery of 3-D coordinates (quantization error, $er{r}_{qu}$), the uncertainty in the determination of the transformation between the camera reference system and the water reference system with the elevation-axis vertical and pointing upward (camera orientation error, $er{r}_{co}$).

As far as the errors $er{r}_{ic}$ and $er{r}_{ec}$ are concerned, the calibration procedures adopted for the analysis of the stereo images here presented followed standard strategies. The internal calibration has been always performed in controlled conditions (laboratory) before the deployment in the field and produced a maximum error of fractional of pixels (<0.5 pixel) that can be considered negligible for all applications. The external calibration was performed using the auto-calibration procedure described in^19^, which has been proven to be as accurate as standard procedures, and for the data here presented provided errors of 0.5 pixel on average. To determine the orientation and displacement between the camera reference system and the still sea water surface, and hence camera orientation error, $er{r}_{co}$, the strategy proposed by^8^ has been adopted in all cases. This method that has been proved to be accurate on field^26,32^ and synthetic^19^ data.

The reliability of the matched pixels is determined by $er{r}_{ma}$, which is governed, on the one hand, by the quality of the calibration procedure and, on the other hand, by the similarity between the left and right images. In general, sharper images are matched with a higher accuracy, as well as accurate matching requires that left- and right- image gray values belong to the same range. These conditions were obtained using small exposure time, around 2 ms at maximum, and gain factor below 30%. Within these limits, those two values were adjusted to fulfill the condition the image histograms have the same median values (around 110–120 for 8-bit images) and span. For difference in median gray values larger than around 10, an histogram matching procedure can be adopted (Fig. 7). A good indicator of the impact of $er{r}_{ma}$ is the number of pixel matched (otherwise discarded by the dense-stereo processing). For the stereo cases here provided most of the image pixels were matched (around 3 millions for 5 Mpixel cameras), with missing correspondences only on the few bright and dark uniform regions (Fig. 8). In addition, occasional mismatches may occur close to the edges of the matched area. To account for those potential errors (resulting in unwanted spikes), for the wave analysis (e.g. space-time spectral or statistical) it is recommended to consider only those points lying in the central part of the matched area.

With the left- and right-image pixel matched, what remains to quantify is the value of the 3-D $er{r}_{qu}$ for each surface point. It depends on the camera cell size and pixel numbers, the focal length, the baseline, the camera reciprocal orientation, and the distance from the stereo-camera system to the scene of interest. An example of the spatial distribution of the quantization error^22^ is shown in Fig. 9, for a stereo camera installation 33-m high.

When other sources of data were available, the stereo video system was compared with other sensors (see the Reference studies provided in Table 1). Figure 10 exemplifies a frequency spectrum $E(f)$ estimated from the stereo video system and a wave gauge at BS in 2011 and 2013. More detailed validation for each stereo video dataset can be found at the acquisition documentation (see^10,12,27,31^).

However, this kind of comparison was not always available for all the data provided in this study. As a consequence, the quantization error is the most direct value to estimate the expected accuracy of the observations. Table 3 reports the root-mean-square (RMS) values of the three components of $er{r}_{qu}$, namely vertical ($RM{S}_{z}$) and 2-D horizontal ($RM{S}_{x}$, $RM{S}_{y}$), and the estimated low-high frequency limits (${f}_{lim}$) for each experiment.

The assessment presented in Table 3 was optimized according to different research objectives. So these results could be adjusted for other study purposes, for example, in most of the cases, the quantization error could be reduced by looking at a smaller region closer to the cameras, and the high and low-frequency limits may be improved by applying different filtering techniques. So the results here presented follows our specific interests, but by providing in this study the raw stereo images and all the processing codes we invite the scientific community to improve this processing according to its own interest.

## Usage Notes

The stereo-image data set is archived at^30^ https://sextant.ifremer.fr/eng, 10.12770/af599f42-2770-4d6d-8209-13f40e2c292f. Data provided in this repository are organized according to the station and year of the experiment (e.g. *BS*_2011, *BS*_2013, *AA*_2014 …). Each record is characterized by the time that the acquisition starts and the frame rate in Hz, e.g. *BS*_2011/2011–10–01_16–18–00_15 *Hz*. The following data are publicly available there:

1. The raw uncompressed images (8 bit gray scale Tagged Image File TIF or Portable Network Graphics PNG formats) for the synchronized left and right stereo cameras. These images are stored in “input” folder. “NNNNNN_01.tif, .png” correspond to left camera images, “NNNNNN_02.tif, .png” correspond to the right camera images and NNNNNN is the sequential image number since the record start.

2. The WASS stereo processing configuration files used to analyse images are stored in “config” folder: distortion_00.xml and distortion_01.xml contains the lens distortion; intrinsics_00.xml and intrinsics_01.xml correspond to the intrinsic cameras calibration parameters; matcher_config.txt and stereo_config.txt are the WASS pipeline configuration files. For more detail information on how to modify and use these files see^9^.

3. A subset of post-processed sea surface elevation space-time fields bi-linearly interpolated on a regular 2-D horizontal uniform grid is stored as NetCDF 4 format file inside the “nc” folder. Decoding information and metadata are included in each file.

Here we provide a full dataset for twelve stereo video records, including examples of post-processed Netcdf outputs, the raw images and the link to the codes repository. So the users can either work with the surface elevation map samples or download the images and reprocess it according to their needs.

## Data Availability

All the data processed in this dataset used the WASS (“*An open-source pipeline for 3-D stereo reconstruction of ocean waves*”) code version 1.4 provided by^9^ and available at http://www.dais.unive.it/wass (the WASS is a full-frame and dense-matching evolution of the previous stereo code developed by^8,26^ and further refined by^10^). This tool is able to (i) reconstruct the sea surface by means of a dense cloud of 3-D points from stereo images, (ii) compute the extrinsic parameters of the stereo rig without the need of tedious calibration on-the field, and (iii) provide the parameters necessary to transform the wave data to a local geographical reference system. The fast 3-D dense stereo reconstruction is based on the OpenCV library^29^ and analyzes the photometrically distinctive features of the water surface to eventually provide the 3-D surface elevation map. When the stereo images are processed by WASS, the generated 3-D point cloud is re-sampled in a uniform grid and the final space-time surface elevation is stored in a standard NetCDF4 file. We developed an additional tool (https://github.com/fbergama/wassncplot) that provides the mapping between each image pixel (either from left or right camera) to the 3-D geographical reference frame of the grid stored in the NetCDF4 file. This way, any further processing can take into account both the luminance of each pixel and its 3-D location onto the sea-surface. Possible applications include the analysis of white-capped areas, the 3-D tracking of floating objects and measuring the shape and extent of individual waves. Note that this mapping is in general one-way (ie. from image to 3-D space) since cameras are angled with respect to the sea-surface and some 3-D points in the back-side of the waves are occluded by the crests. In this context, the *wassncplot* tool computes the correct intersection between the 3-D ray in space passing through the camera center and a pixel $p$ with the gridded surface. Since multiple intersections may occur, only the one nearest to the camera is considered for the mapping. Operatively, the operation is performed by projecting each triangle of the grid to the image plane corresponding to one of the two stereo cameras. The coordinate of each grid vertex is interpolated considering the barycentric coordinates within each triangle corrected by the depth of the point. A Z-buffer is used to discard occluded points and produce the final mapping as a 3-dimensional data cube $D$ of size ($W\times H\times 3$) in which the first two dimensions spans the image space and the third the 3-D coordinates of a point (ie. ${D}_{i,j,0},{D}_{i,j,1},{D}_{i,j,2}$ are the $x,y,z$ coordinates of the 3-D point corresponding into the pixel *i*, *j* in the image). Optionally, the tool can produce a rendering of the 3-D surface grid on top of the original images for a qualitative evaluation of the reconstructed data (See Fig. 6).
